# Risk stratification and multi-omics reveal RAD51 inhibition as a radiosensitizing strategy in triple-negative breast cancer

**DOI:** 10.3389/fimmu.2026.1866358

**Published:** 2026-07-28

**Authors:** Ran An, Jianming Li, Tianhui Liu, Cunyu Tang, Hong Lu

**Affiliations:** 1 Department of Breast Imaging, Tianjin Medical University Cancer Institute and Hospital, National Clinical Research Center for Cancer, Tianjin, China; 2 Tianjin Medical University Cancer Institute & Hospital, Tianjin’s Clinical Research Center for Cancer, Tianjin, China; 3 Key Laboratory of Breast Cancer Prevention and Therapy, Tianjin Medical University, Ministry of Education, Tianjin, China; 4 Tianjin Medical University Cancer Institute & Hospital, Key Laboratory of Cancer Prevention and Therapy, Tianjin, China; 5 Department of Radiation Oncology, Tianjin Medical University Cancer Institute and Hospital, National Clinical Research Center of Cancer, Tianjin, China

**Keywords:** machine learning, RAD51, radioresistance, single-cell RNA sequencing, triple-negative breast cancer

## Abstract

**Background:**

Triple-negative breast cancer (TNBC) is a highly aggressive subtype characterized by significant inter- and intra-tumoral heterogeneity. Its varied response to radiotherapy limits clinical outcomes, yet the underlying molecular determinants remain poorly understood.

**Methods:**

By integrating single-cell and bulk transcriptomic data, we profiled cell-type-specific radiosensitivity and defined distinct molecular subtypes via consensus clustering. A random survival forest (RSF) prognostic model was then constructed using 34 survival-associated genes and interpreted via SHAP analysis, and its performance was validated in multiple independent external cohorts. RAD51 was prioritized via Dependency Map (DepMap) analysis, and its functional role was validated *in vitro* and *in vivo*, supporting its role as a biological dependency in radiotherapy response. Moreover, tumor immune remodeling was further evaluated by immunofluorescence and protein analysis.

**Results:**

Single-cell analysis revealed that fibroblasts, macrophages, and proliferative cells harbored elevated radioresistance signatures compared to lymphoid lineages. Bulk profiling identified two distinct subtypes: Cluster A (immunosuppressive, radioresistant, poor prognosis) and Cluster B (immune-active, favorable survival). The RSF model robustly stratified patient risk, with high-risk tumors tightly linked to an immune-desert microenvironment. DepMap analysis nominated RAD51 as a core biological dependency. Importantly, RAD51 knockdown sensitized TNBC cells to irradiation by exacerbating DNA damage, triggering G2/M phase arrest, and driving apoptosis. Consistently, *in vivo* RAD51 silencing synergized with radiotherapy to significantly suppress tumor growth. Notably, this combination reshaped the immunosuppressive TME, driving robust CD8^+^ T cell infiltration, M1 macrophage repolarization, and adaptive PD-L1 upregulation.

**Conclusion:**

This integrative multi-omics study delineates the cellular and molecular landscape of TNBC radioresistance. The RSF model provides a robust tool for risk stratification, while identifying RAD51 as a potential therapeutic target to restore radiosensitivity and reverse immunosuppression, particularly in high-risk, immune-cold tumors.

## Introduction

Breast cancer remains one of the most common malignancies and a major cause of cancer-related death among women worldwide, with an increasing incidence observed in younger patients in several populations (1–3). TNBC, which lacks expression of estrogen receptor (ER), progesterone receptor (PR), and human epidermal growth factor receptor 2 (HER2), accounts for approximately 15-20% of breast cancer cases (4–6). Clinically, TNBC is characterized by aggressive biology, late-stage diagnosis, and high metastatic potential. Because it lacks established actionable receptors, systemic management still relies heavily on chemotherapy, and outcomes remain inferior to those of other breast cancer subtypes (7–9).

In the multimodal treatment of TNBC, radiotherapy serves as a crucial modality for local control, effectively reducing the risk of recurrence and improving survival in selected patients (10–14). However, radiotherapy response is highly heterogeneous across patients (15) and even among different cellular compartments within the same tumor (16). Large-scale population data suggest that radiotherapy is associated with an increased risk of second primary malignancies, particularly in younger patients with long-term follow-up, underscoring the need to optimize patient selection and therapeutic strategies (17). Furthermore, radiosensitivity differs by molecular subtype; for example, HER2-positive tumors often exhibit radioresistance, whereas TNBC may derive substantial mortality benefit from radiotherapy in certain clinical contexts (18). These observations highlight an unmet need to define robust determinants of radiosensitivity and radioresistance in TNBC.

Mechanistically, radioresistance arises from both tumor-intrinsic and tumor-extrinsic factors (19). Tumor cells may evade radiotherapy through enhanced DNA damage repair, altered cell-cycle checkpoints, and stem-like properties, whereas the tumor microenvironment (TME) can shape therapeutic response by remodeling vascular, stromal, and immune ecosystems following irradiation (19–23). Radiotherapy can induce dynamic TME reprogramming that promotes immune evasion and recurrence, with cancer-associated fibroblasts (CAFs), tumor-associated macrophages (TAMs), and myeloid-derived suppressor cells (MDSCs) acting as key mediators of immunosuppression and resistance (24–26). Therefore, dissecting cell-type-specific radiosensitivity and its interplay with the immune microenvironment is essential for identifying predictive biomarkers and rational combination strategies.

Single-cell RNA sequencing (scRNA-seq) provides a high-resolution view of intratumoral heterogeneity and tumor–immune interactions; however, single-omic analyses alone are often insufficient to explain inter-patient variability in radiotherapy response or to yield clinically actionable predictors. To address this gap, we integrated bulk RNA-seq data from multiple cohorts to derive a radiosensitivity-associated gene panel. Using consensus clustering and scRNA-seq validation, we characterized the expression landscape of these genes across molecular subtypes and resolved their cellular heterogeneity within TNBC.

Building on this foundation, the present study employed a multi-algorithmic approach to develop an RSF prognostic model for TNBC based on 34 survival-associated genes, and applied SHAP interpretation to stratify tumors into clinically distinct immune-active and immune-desert phenotypes. To identify potential vulnerabilities embedded within this signature in an unbiased manner, we further interrogated the DepMap database, which prioritized RAD51 as the most critical dependency, and subsequently validated its functional role through *in vitro* and *in vivo* experiments. Rather than reiterating the well-characterized DNA-repair function of RAD51, our framework was designed to nominate such dependencies in a data-driven manner and to interpret them within the immune context of TNBC. Collectively, this multi-layered approach integrates machine learning and transcriptomic analyses to delineate gene-expression profiles associated with radiotherapy response in TNBC, thereby offering mechanistic insight into radioresistance and nominating candidate targets for future therapeutic development.

## Materials and methods

### Data acquisition

scRNA-seq data of TNBC were retrieved from the TISCH database (GSE148673, GSE161529). Bulk transcriptomic profiles and clinical data were obtained from The Cancer Genome Atlas (TCGA)-BRCA (140 cases) and GSE58812 (107 cases), and four independent external validation cohorts (GSE20685, GSE9893, GSE7390, and GSE42568). A list of 395 radiation resistance-associated genes was sourced from DbCESR (27).

### Single-cell RNA-seq processing and cell-cell communication

Quality control retained cells with 200–6, 000 genes, 1, 000–20, 000 unique molecular identifier (UMIs), and <20% mitochondrial transcripts. Data were normalized, log-transformed, and subjected to principal component analysis (PCA). Batch effects were corrected using Harmony, followed by Uniform Manifold Approximation and Projection (UMAP) visualization. Cell types were annotated based on Tumor Immune Single-cell Hub (TISCH) references. Marker genes were identified using FindAllMarkers and COSG, followed by KEGG/Reactome enrichment via clusterProfiler.

Intercellular communication networks, including pathway-specific interactions and incoming/outgoing signaling strengths, were inferred from normalized expression matrices using CellChat.

### Radiosensitivity scoring and abundance analysis

Single-cell Radiosensitivity Gene Scores (RSGS) and Hallmark pathway scores were calculated using GSVA. Cell abundance shifts between high- and low-RSGS groups (dichotomized by median) were comprehensively evaluated by integrating direct proportions, miloR-based differential abundance (SpatialFDR control), and Fisher’s exact test odds ratios (ORs) (28). These metrics were Z-score normalized and summed to yield a composite resistance association score.

### Bulk RNA-seq analysis and molecular subtyping

TCGA and GSE58812 cohorts were merged (16, 915 common genes). Univariate Cox regression identified 34 survival-associated genes (p<0.05), which were subjected to unsupervised consensus clustering (ConsensusClusterPlus; PAM, Pearson distance, 1000 iterations). Biological features were characterized via GSVA (Hallmark, KEGG, Reactome), ssGSEA (26 StemChecker signatures), and maftools (mutational/CNV profiling). Immune infiltration was quantified using IOBR, integrating MCPcounter, EPIC, CIBERSORT, IPS, quanTIseq, ESTIMATE, and TIMER.

### Machine learning-based prognostic modeling

Ten machine learning algorithms (RSF, least absolute shrinkage and selection operator (LASSO), survival support vector machine (Survival-SVM), supervised principal components (SuperPC), ridge, partial least squares regression for Cox (plsRcox), CoxBoost, Stepwise Cox, elastic net) and their combinations were evaluated using the 34 prognostic genes. The model was trained on the discovery cohort, and the locked optimal model was subsequently applied without re-fitting to the four independent external cohorts for validation. Model performance was benchmarked via cross-validated C-index, time-dependent ROC, and Kaplan-Meier analyses. The optimal RSF model was interpreted using SHAP values. Gene essentiality was assessed via the DepMap.

### Cell culture and transfection

Murine 4T1 and human MDA-MB-231 TNBC cells (ATCC) were cultured in RPMI-1640/DMEM supplemented with 10% FBS and 1% penicillin/streptomycin. Lentiviral vectors encoding shRNA against RAD51 (sh-RAD51) or a scramble control (NC) (Gene Adv, China) were transduced with polybrene (8 μg/mL), followed by puromycin selection (2 μg/mL, 5 days).

### Western blot analysis

Proteins (30μg), extracted using sodium dodecyl sulfate (SDS) lysis buffer supplemented with 1x protease (Sigma-Aldrich, USA) and 1x phosphatase (Bimake, USA) inhibitors, were separated by SDS-PAGE and transferred to PVDF membranes. After blocking with 5% non-fat milk, membranes were probed with primary antibodies against RAD51 (CST, #84555), PD-L1 (Abmart, #T55980) and GAPDH (CST, #92310) overnight at 4 °C. Signals were detected using an ECL kit.

### Clinical tissue samples and immunohistochemistry

Twenty FFPE TNBC surgical specimens from patients receiving adjuvant RT underwent standard IHC. Sections were deparaffinized, rehydrated, antigen-retrieved, and incubated with anti-RAD51 (CST, #84555). Signals were developed using a DAB substrate, and nuclei were counterstained with hematoxylin.

### Immunofluorescence staining

For *in vitro* IF, irradiated cells on coverslips were exposed to 8 Gy of irradiation and cultured for 12 hours. Subsequently, the irradiated cells were fixed, permeabilized (0.3% Triton X-100), and stained with anti-γ-H2AX (CST, #60566) and DAPI. Mean Fluorescence Intensity (MFI) was quantified using ImageJ.

### Clonogenic survival assay

Cells (1, 000/well) were seeded into 6-well plates, exposed to varying doses of irradiation (0–8 Gy), and cultured for 7–14 days. Surviving colonies (>50 cells/colony) were fixed with 4% paraformaldehyde (PFA), stained with 0.1% crystal violet, and manually counted to calculate the survival fraction.

### EdU cell proliferation assay

Cell proliferation was evaluated using an EdU Proliferation Kit (Beyotime). Treated cells were incubated with 10 μM EdU for 2 h, fixed, permeabilized, and stained according to the manufacturer’s protocol. Nuclei were counterstained with DAPI, and EdU-positive proliferating cells were quantified via fluorescence microscopy.

### Flow cytometry for cell cycle and apoptosis

For cell cycle analysis, treated cells were harvested at 48 hours post-irradiation (8 Gy), washed with cold PBS, and fixed in 70% ice-cold ethanol overnight at -20 °C. Cells were then treated with RNase A and stained with Propidium Iodide (PI) for 30 minutes in the dark.

For apoptosis analysis, cells were harvested at 48 hours post-irradiation (8 Gy) and double-stained using an Annexin V-FITC/PI Apoptosis Detection Kit (catalog No.559763, BD) following the manufacturer’s protocols. All flow cytometric acquisitions were performed on Beckman flow cytometer, and data were analyzed using FlowJo software.

### *In vivo* tumor growth experiments and histology

Female BALB/c mice (6 weeks) were subcutaneously injected with 5×10^5^ 4T1 cells (NC or sh-RAD51). When tumors approximately reached 100 mm³, mice (n=7/group) were randomized to: NC, sh-RAD51, RT alone (8 Gy focal radiation), and sh-RAD51+RT. To ensure proper positioning and immobilization during localized irradiation, mice were maintained under continuous 2% isoflurane anesthesia. Tumor volumes (L×W^2^/2) were monitored every 3 days. At the end of the observation period, all mice were humanely euthanized via cervical dislocation under deep anesthesia (via intraperitoneal injection of 250 mg/kg tribromoethanol). Excised tumors were FFPE-processed for Ki-67 IHC (Abcam, #ab833) and TUNEL fluorescence staining (Beyotime). Additionally, to evaluate the remodeling of the immune microenvironment, tumor sections were subjected to immunofluorescence staining for CD8^+^ T cells (CST, #40345) and multiplex immunofluorescence for macrophage polarization, M1 marker CD86 (Abcam, #ab305361) and M2 marker CD206 (Abcam, #ab300621).

### Drug sensitivity analysis

Drug IC50 values were predicted using oncoPredict, highlighting the top 20 differentially sensitive compounds between RSF-defined high- and low-risk groups.

### Statistical analysis

Bioinformatic analyses were conducted in R (v4.2.2). Continuous and categorical variables were compared using Wilcoxon rank-sum and Chi-square tests, respectively. Survival was assessed via Kaplan-Meier (log-rank) and Cox models. Experimental data, analyzed via GraphPad Prism (v9.0.2), are presented as mean ± SEM from ≥3 replicates. Group comparisons utilized two-tailed Student’s t-tests or ANOVA. P < 0.05 was considered significant.

## Results

### Single-cell analysis reveals TME heterogeneity linked to radioresistance programs

**Figure 1:**
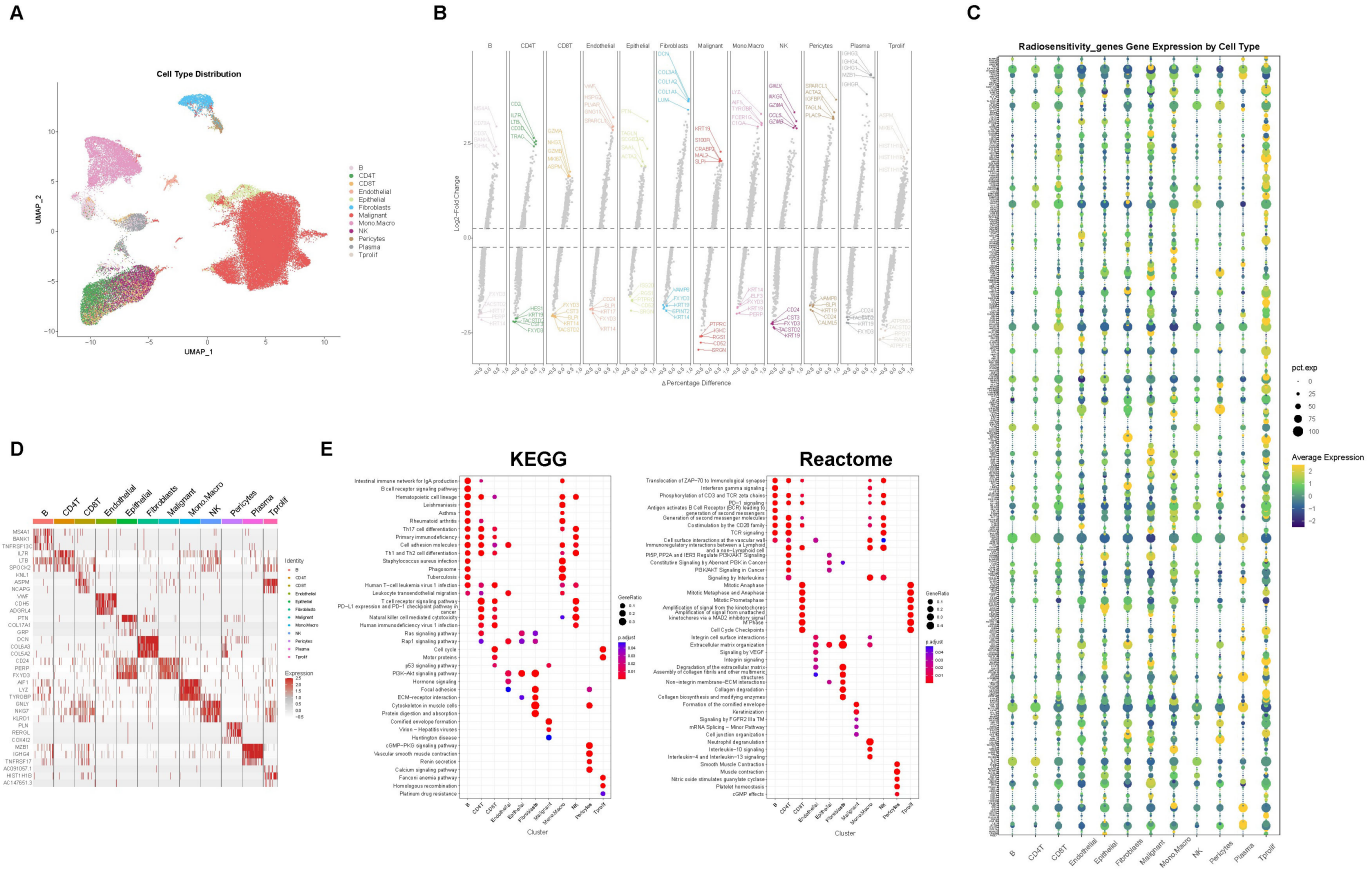
Single-cell analysis reveals tumor cell heterogeneity and its association with radioresistance. **(A)** Dimensionality reduction of single-cell data showing cell clustering; cell-type annotation referenced from the TISCH database. **(B)** Differentially expressed genes identified across cell types using the FindAllMarkers function; top 5 up- and down-regulated genes are shown for each cell type. **(C)** Expression levels of radiotherapy-tolerance–related genes across cell subpopulations. **(D)** Cell-type marker genes identified by the COSG R package; heatmap displays the top 3 markers per cell type. **(E)** Pathway enrichment analysis based on COSG-derived markers: the top 100 genes per cell type were subjected to enrichment (clusterProfiler), with KEGG and Reactome results displayed.

To investigate the cellular heterogeneity within the TME of TNBC and its impact on radiotherapy response, we analyzed scRNA-seq data. Using TISCH database annotations, cells were classified into distinct subpopulations (**Figure 1A**), each exhibiting specific gene expression profiles (**Figure 1B**). Literature-curated radioresistance genes were unevenly distributed across these subsets, being notably enriched in proliferative T cells (Tprolifs) (**Figure 1C**) (27). Marker genes for each subpopulation were visualized (**Figure 1D**), and KEGG/Reactome enrichment indicated their involvement in diverse biological processes, including cell cycle, DNA repair, and energy metabolism (**Figure 1E**). Furthermore, radiosensitivity significantly correlated with multiple Hallmark pathways, such as G2/M checkpoints, E2F targets, and glycolysis (**Supplementary Figure 1**), indicating that TNBC radioresistance is driven by both intrinsic tumor cell states and TME functional activity.

### Radiosensitivity scoring distinguishes cellular subpopulations in the TME

**Figure 2:**
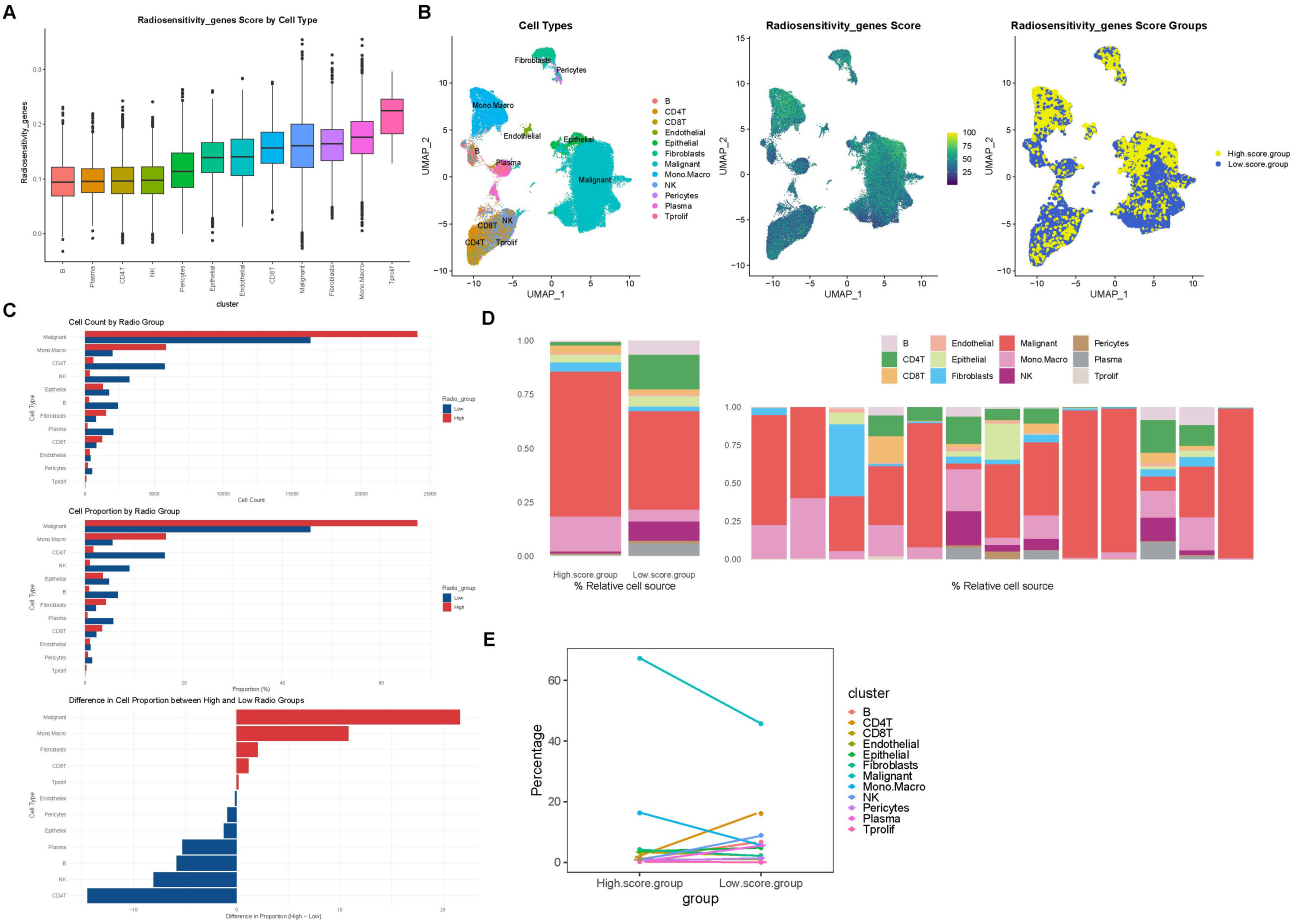
Radiosensitivity scoring reveals cellular subpopulation heterogeneity in the tumor microenvironment. **(A)** Radiosensitivity scores across cell types. **(B)** UMAP visualization of radiosensitivity gene-set scores; cells are stratified into high- and low-score groups. **(C)** Differences in cell abundance between high- and low-score groups: (top) cell counts; (middle) cell proportion; (bottom) proportion differences (high-low). **(D)** Relative cell source within each sample, compared between high- and low-score groups. **(E)** Trends in cell counts across cell types between high- and low-score groups.

We next constructed a RSGS and projected it onto the single-cell atlas to compare radiotherapy-associated programs across cell types. RSGS varied substantially among populations (**Figure 2A**): Tprolifs, monocytes/macrophages (Mono.Macros), fibroblasts, and malignant cells exhibited higher scores, whereas B cells, plasma cells, CD4^+^ T cells, and NK cells showed lower scores. UMAP visualization segregated cells into high- and low-score groups based on median RSGS (**Figure 2B**). Analyzing absolute counts andrelative proportions confirmed that malignant cells and Mono.Macros dominated the high-score group, while immune cells were abundant in the low-score group (**Figure 2C**). The robustness of this RSGS distribution was consistently validated across individual samples and shifting cell-number trends (**Figures 2D, E**), suggesting that malignant and stromal lineages primarily mediate radioresistance.

### Abundance shifts and cell–cell communication differ between high- and low-RSGS states

**Figure 3:**
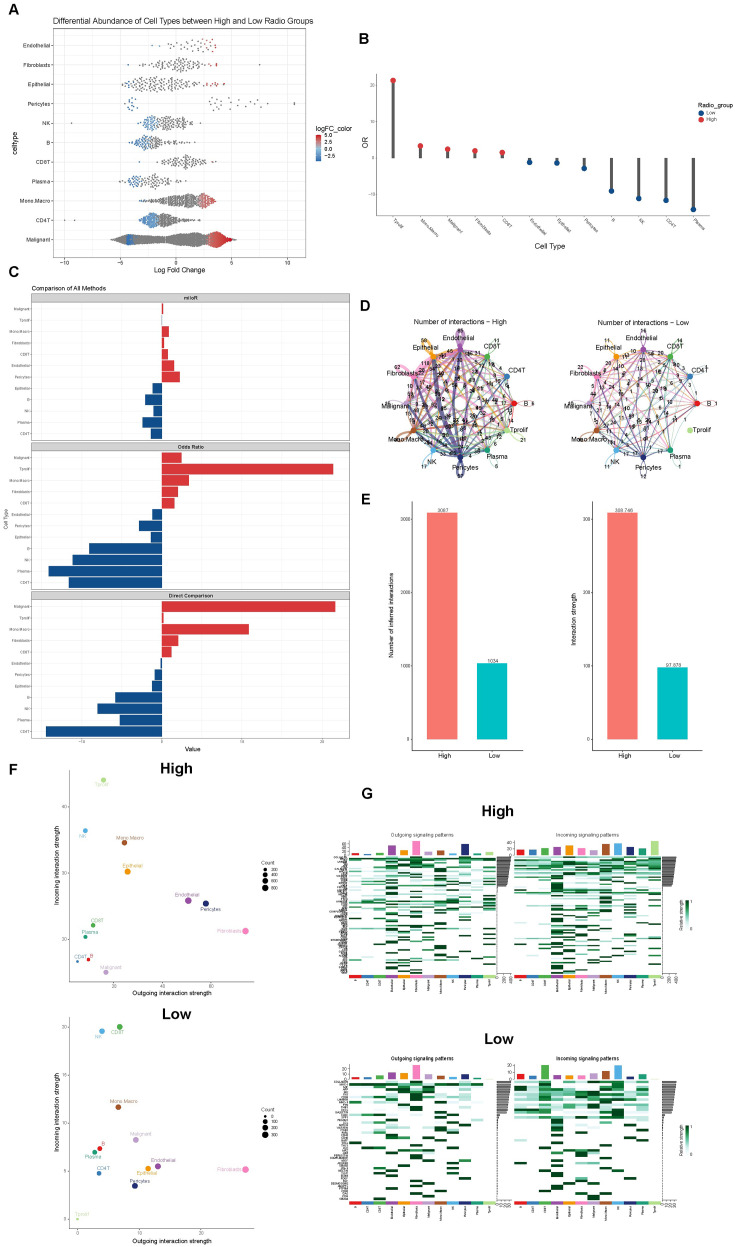
Cellular composition and intercellular communication associated with radiosensitivity scoring. **(A)** Fold-change in cell counts between high- and low-radiosensitivity score groups. **(B)** OR distribution between high- and low-score groups. Odds ratio (OR; Ro/e) analysis by contingency tables built from metadata, with Fisher’s exact test yielding OR and P values. To standardize directionality, ORs were assigned negative for the low-score group and positive for the high-score group. **(C)** Concordance across three methods and unified directionality: Direct comparison: difference in cell-type fractions (High-Low); positive indicates enrichment in High. miloR (differential abundance, DA): neighborhood-level DA with contrast “NIHigh-NILow”; logFC > 0 indicates High enrichment; SpatialFDR controls significance. OR (Ro/e): 2×2 tests per cell type vs. High/Low; code assigns negative to Low and positive to High so OR > 0 indicates High enrichment and OR < 0 indicates Low enrichment. **(D)** Cell–cell communication analysis contrasting High vs. Low score groups. **(E)** Comparison of the total number of interactions and overall interaction strength between groups. **(F)** Scatter plot of interaction strengths among cell types in the High-score group, highlighting outgoing and incoming signaling intensities (high vs. low). **(G)** Heatmaps showing pathway-level outgoing (left) and incoming (right) signaling strengths across cell types.

To further quantify compositional differences, we assessed abundance shifts using log fold change and odds ratios. Malignant cells and Mono.Macros were enriched in the high-RSGS group, whereas B cells, CD4^+^ T cells, NK cells, and plasma cells were enriched in the low-RSGS group (**Figures 3A, B**). A composite score integrating log fold change (logFC), OR, and direct differences conclusively mapped specific lineages to radiosensitivity states (**Figure 3C**; **Supplementary Figure 2**). Cell-cell communication analysis revealed a denser interactome in the high-score group, characterized by increased total interactions and aggregate signaling strength (**Figures 3D, E**), alongside distinct information flow patterns (**Supplementary Figure 3**). Notably, fibroblasts and endothelial cells acted as dominant signal senders in the high-score group, whereas the low-score group exhibited an immune-centered, receiver-dominant landscape (**Figure 3F**). These interactome divergences were further quantified across pathways (**Figure 3G**), highlighting a stroma-driven multicellular network in radioresistant TNBC.

### Bulk RNA-seq defines two prognostic subtypes with distinct transcriptional and clinical features

**Figure 4:**
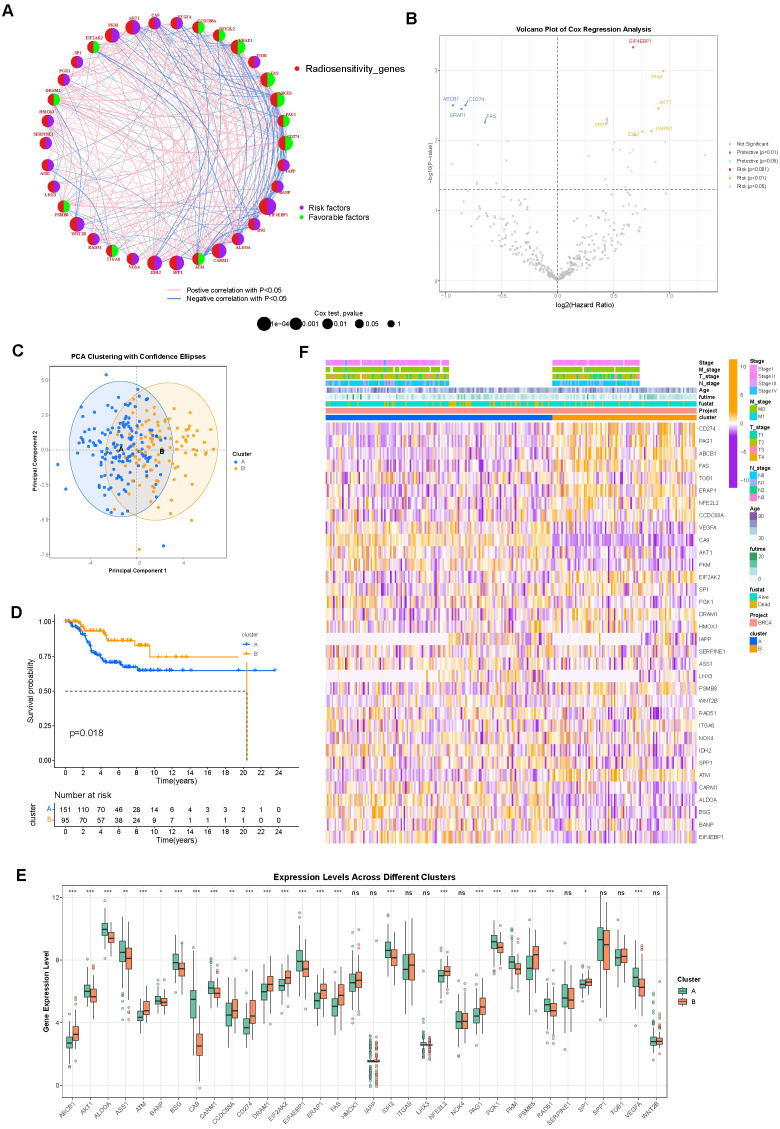
Bulk RNA-seq identifies two prognostic subtypes with distinct expression and survival profiles. **(A)** Two bulk RNA-seq cohorts were merged (16, 915 genes; 247 samples; 246 with follow-up > 0). Of 395 radiotherapy-tolerance-related genes, 378 were detected and subjected to correlation and univariate Cox analyses (R packages survival, survminer; visualization with ggplot2). In the network plot, purple indicates risk factors and green indicates protective factors (circle size reflects P value); edges denote significant correlations between metabolism-related pathways (P < 0.05). From 340 detectable tolerance genes, 34 showed Cox P < 0.05 and were selected for subtyping. **(B)** The volcano plot showing the results of the Cox regression analysis. **(C)** PCA plot illustrating the separation of samples by the two subtypes. **(D)** Kaplan-Meier survival analysis comparing overall survival between the two subtypes. **(E)** Expression profiles of the 34 prognostic genes across the two subtypes. **(F)** Heatmap integrating clinical features, gene expression, and subtype labels to depict their associations. ns (p > 0.05), * (p < 0.05), ** (p < 0.01), *** (p < 0.001).

In bulk transcriptomic cohorts, we integrated datasets to obtain 247 TNBC samples (**Figure 4A**). Among curated radioresistance-related genes, 34 were associated with overall survival by correlation screening and univariate Cox analysis (p<0.05) and were used for subtype discovery (**Figure 4B**). Unsupervised consensus clustering based on these genes robustly stratified patients into Cluster A and Cluster B (**Supplementary Figure 4**), showing clear separation in principal component analysis (**Figure 4C**). Cluster B exhibited a significant survival advantage over Cluster A (**Figure 4D**). Differential expression analysis confirmed distinct molecular patterns between the clusters (**Figure 4E**), with Cluster A strongly associated with advanced clinical stages (III-IV) and older age (**Figure 4F**).

**Figure 5:**
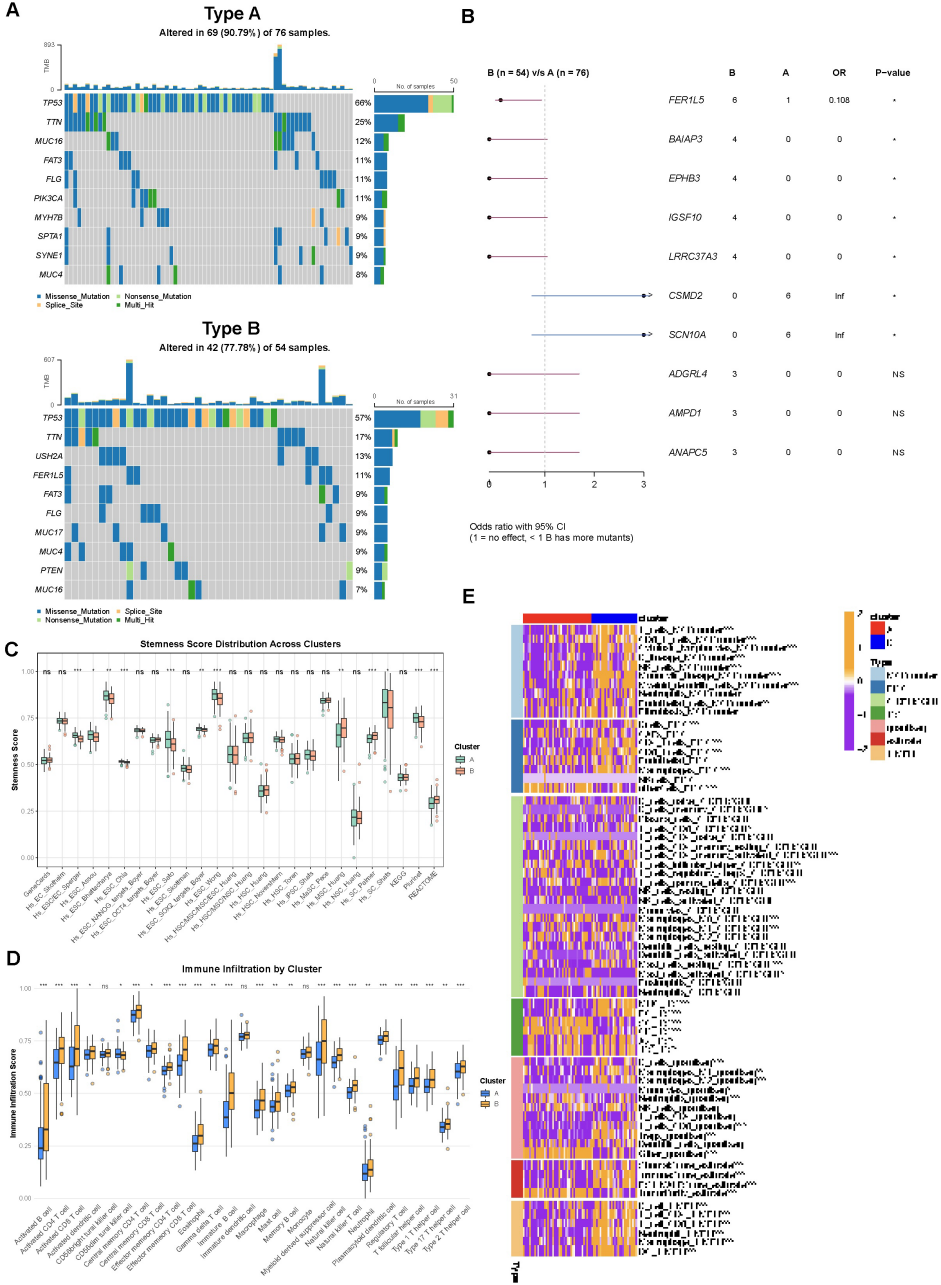
Subtype-level contrasts: integrated pathway enrichment, mutation landscape, stemness, and immune infiltration. **(A)** Somatic mutation landscapes for Cluster A and Cluster B generated with maftools R package. **(B)** Comparison of gene mutation differences between the two clusters using maftools R package. **(C)** Stemness evaluation based on ssGSEA scores from stemness-related signatures. The 26 stem gene sets acquired from StemChecker database. **(D)** Immune infiltration comparison between clusters using GSVA-derived ssGSEA scores; significance indicated as ns (p > 0.05), * (p < 0.05), ** (p < 0.01), *** (p < 0.001). **(E)** Heatmap showing the relationships among subtypes, gene expression, and the immune microenvironment; immune cell infiltration estimated with the IOBR package across seven methods (MCPcounter, EPIC, CIBERSORT, IPS, quanTIseq, ESTIMATE, TIMER).

Subtype-level pathway analysis across multiple databases (KEGG, Reactome, Hallmark) revealed that Cluster A was enriched in cell cycle and DNA repair pathways, whereas Cluster B was driven by immune activation and antigen presentation (**Supplementary Figure 5**). Genomically, Cluster A bore a higher mutational burden (e.g., TP53 mutations) and higher stemness scores (**Figures 5A–C**). Conversely, microenvironmental deconvolution confirmed that Cluster B harbored extensive effector immune cell infiltration (**Figures 5D, E**), representing an immune-hot, prognostically favorable phenotype.

### An RSF-based 34-gene model robustly predicts prognosis and aligns with clinical risk

**Figure 6:**
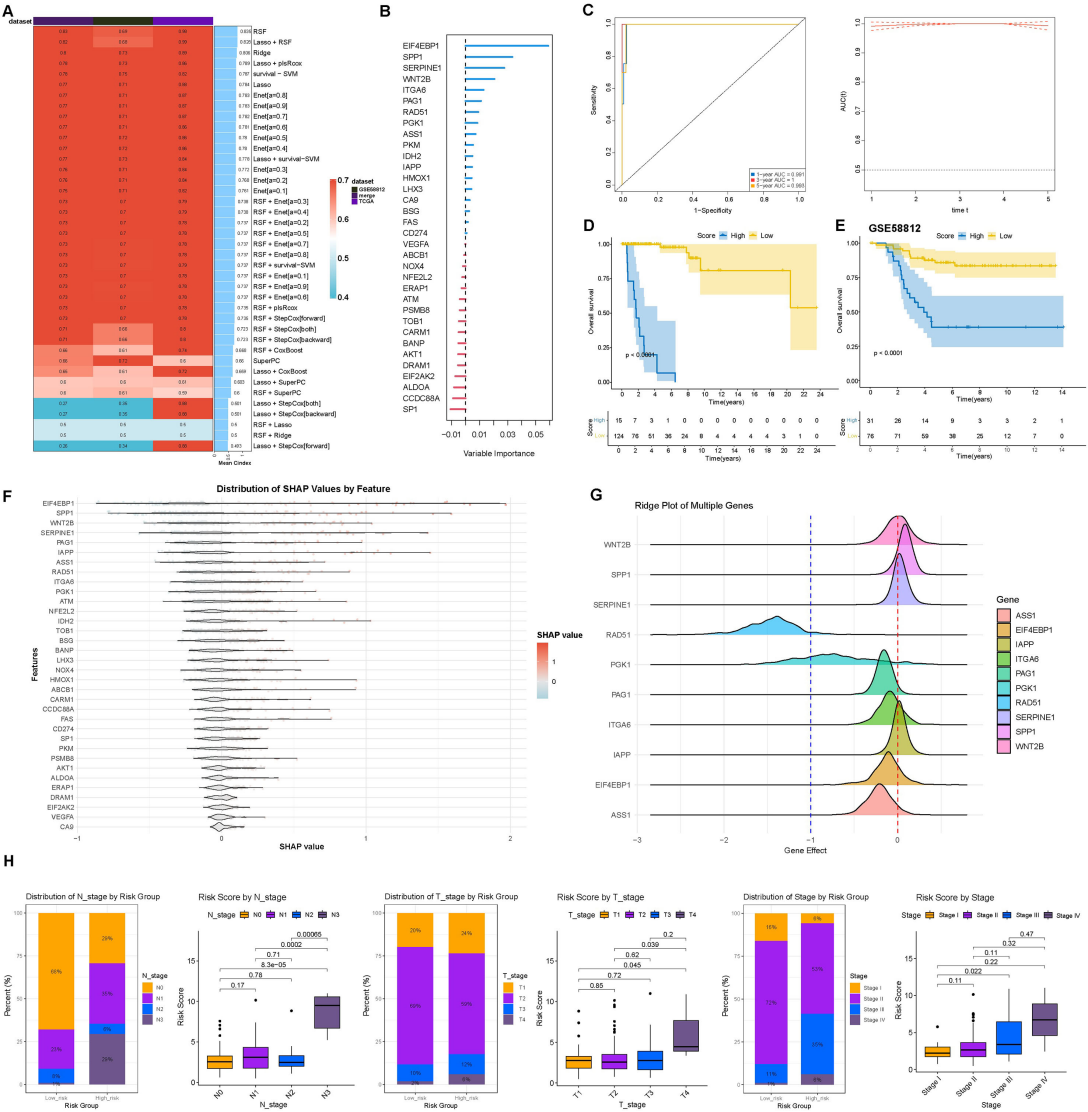
A radioresistance-gene–based RSF model predicts prognosis and stratifies clinical risk. **(A)** Prognostic modeling based on 34 genes using ten algorithms (RSF, LASSO, Survival-SVM, SuperPC, Ridge, plsRcox, CoxBoost, Stepwise Cox, elastic network (Enet)) and their combinations; resulting C-indexes are summarized. **(B)** RSF model feature importance ranking of modeling genes. **(C)** Prognostic performance in TCGA using the RSF model, including time-dependent ROC curves. **(D)** Kaplan-Meier survival curves in TCGA stratified by RSF risk score. **(E)** Kaplan-Meier survival curves in GSE58812. **(F)** SHAP analysis quantifying the contribution of radiotherapy-tolerance–related genes to RSF predictions. **(G)** DepMap analysis of the top 10 SHAP-ranked genes; ridgeline plots show dependency score distributions. **(H)** Associations between RSF risk score and clinical features: left, N stage; middle, T stage; right, overall stage.

Benchmarking 10 machine-learning algorithms demonstrated that the RSF model achieved the highest concordance index (mean C-index = 0.835) (**Figure 6A**), with variable importance analysis identifying key contributors (**Figure 6B**; explicitly detailed in **Supplementary Table 1**). In TCGA, the RSF model exhibited strong predictive accuracy (**Figure 6C**). The RSF-derived risk score (RS) stratified patients into high- and low-risk groups with significantly different outcomes in the training cohort, the validation cohort, and the combined dataset (**Figures 6D, E**, **Supplementary Figure 6A**). Then we applied the established model to independent external breast cancer cohorts. Consistent with our internal cohorts, the model maintained excellent prognostic capability, robustly stratifying patient overall survival across all four independent datasets (**Supplementary Figure 6B**). SHAP interpretability analysis highlighted RAD51, EIF4EBP1, and SPP1 as top contributors to the RS (**Figure 6F**), which aligned with DepMap CRISPRi data showing their essentiality for tumor cell viability (**Figure 6G**). Clinically, RS remained an independent prognostic factor in multivariable models and correlated with advanced clinicopathologic stage (**Supplementary Figures 6C, D**; **Figure 6H**).

### Integrated multi-omics and experimental validation confirm RAD51 mediates TNBC radioresistance

**Figure 7:**
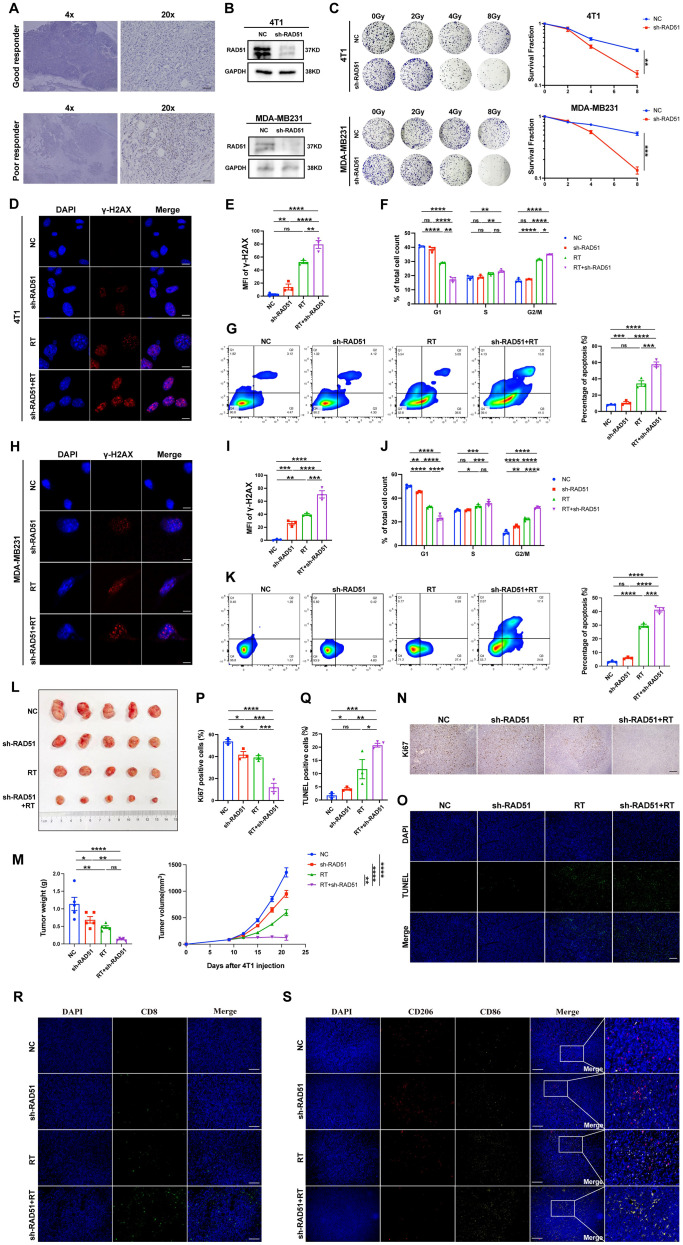
Integrated omics analyses and experimental validation identify RAD51 as a core driver of radioresistance. **(A)** Representative RAD51 IHC images in TNBC surgical specimens (n=20) post-adjuvant RT. Good responders: TRG 0-3; Poor responders: TRG 4-5. Scale bars: 20 μm. **(B)** Western blot validation of RAD51 knockdown (sh-RAD51) vs. negative control (NC) in 4T1 and MDA-MB-231 cells. **(C)** Clonogenic survival of indicated cells across varying radiation doses (0, 2, 4, 8 Gy), normalized to 0 Gy controls. **(D, E, H, I)** Representative immunofluorescence images **(D, E)** and MFI quantification **(H, I)** of γ-H2AX foci across indicated treatments. Scale bars: 200 μm. **(F, J)** Flow cytometric analysis of cell-cycle distribution (G1/S/G2-M) post-treatment. **(G, K)** Annexin V/PI flow cytometry plots and quantification of total apoptosis. **(L, M)** Tumor growth curves **(L)** and terminal tumor weights **(M)** in the 4T1 subcutaneous model across the four cohorts. **(N–Q)** Representative images and quantification of Ki-67 and TUNEL staining in tumor tissues. **(R, S)** Representative immunofluorescence images of CD8^+^, CD206^+^, and CD86^+^ cell infiltration in tumor tissues. Scale bars: 20 μm. Statistical analysis: Data are presented as mean ± SEM. Significance was assessed by Student’s t-test **(C)**, one-way ANOVA **(E, G, I, K, M, P, Q)**, two-way ANOVA **(F, J)**, or log-rank test. *P < 0.05; **P < 0.01; ***P < 0.001; ****P < 0.0001.

To define biological correlates of RS, we correlated RS with transcriptome-wide expression and performed enrichment analysis of RS-associated genes (**Supplementary Figures 7A–E**), highlighting coordinated immune and stress-response programs linked to risk status. Based on SHAP and DepMap rankings, we prioritized RAD51 for experimental validation. Clinically, RAD51 expression was significantly elevated in tissues from poor radiotherapy responders compared to good responders (**Figure 7A**; **Supplementary Figure 8A**).

Functionally, stable RAD51 knockdown in 4T1 and MDA-MB-231 cells was confirmed by Western blot (**Figure 7B**). RAD51 depletion significantly reduced clonogenic survival after irradiation (0–8 Gy) (**Figure 7C**) and further suppressed post-irradiation proliferation in EdU assays (**Supplementary Figures 8B, C**). Consistent with impaired DNA damage repair, RAD51 knockdown increased residual γ-H2AX foci following irradiation (**Figures 7D, H**). Previous studies indicate that irradiated tumor cells often modulate their cell cycle to adopt a radiation-tolerant persistent state, thereby facilitating survival and subsequent regrowth (29). In our study, RAD51 depletion effectively dismantled this adaptive strategy. Rather than undergoing reversible adaptation, the accumulation of unrepaired DNA double-strand breaks drove these cells into a persistent G2/M arrest (**Figures 7F, J**), ultimately leading to a significant increase in apoptosis (**Figures 7G, K**).

*In vivo*, RAD51 knockdown potentiated radiotherapy-mediated tumor control, yielding reduced tumor volume and weight (**Figures 7L, M**), accompanied by decreased Ki-67 staining and increased TUNEL positivity (**Figures 7N–Q**). Importantly, histological examination of major organs revealed no obvious systemic toxicity or pathological changes across all treatment groups (**Supplementary Figure 8D**). Given that our framework tightly linked the RSF signature to the immune microenvironment, we next examined whether the enhanced radiotherapeutic efficacy in sh-RAD51 tumors was accompanied by remodeling of the TME. Multiplex immunofluorescence revealed that the sh-RAD51 + RT combination provoked a significant infiltration of CD8^+^ T cells and drove macrophage polarization toward an anti-tumor M1 phenotype, alongside a concomitant depletion of immunosuppressive M2 macrophages (**Figures 7R, S**; **Supplementary Figure 8E**). Western blot analysis demonstrated that this therapy-induced immune activation was paralleled by an adaptive upregulation of PD-L1 expression in the residual tumor tissues (**Supplementary Figure 8F**). Together, these results not only validate RAD51 as a key driver of TNBC radioresistance but also highlight its critical role in reversing an immunosuppressive TME following irradiation.

### Risk stratification unveils genomic instability and drug sensitivity

**Figure 8:**
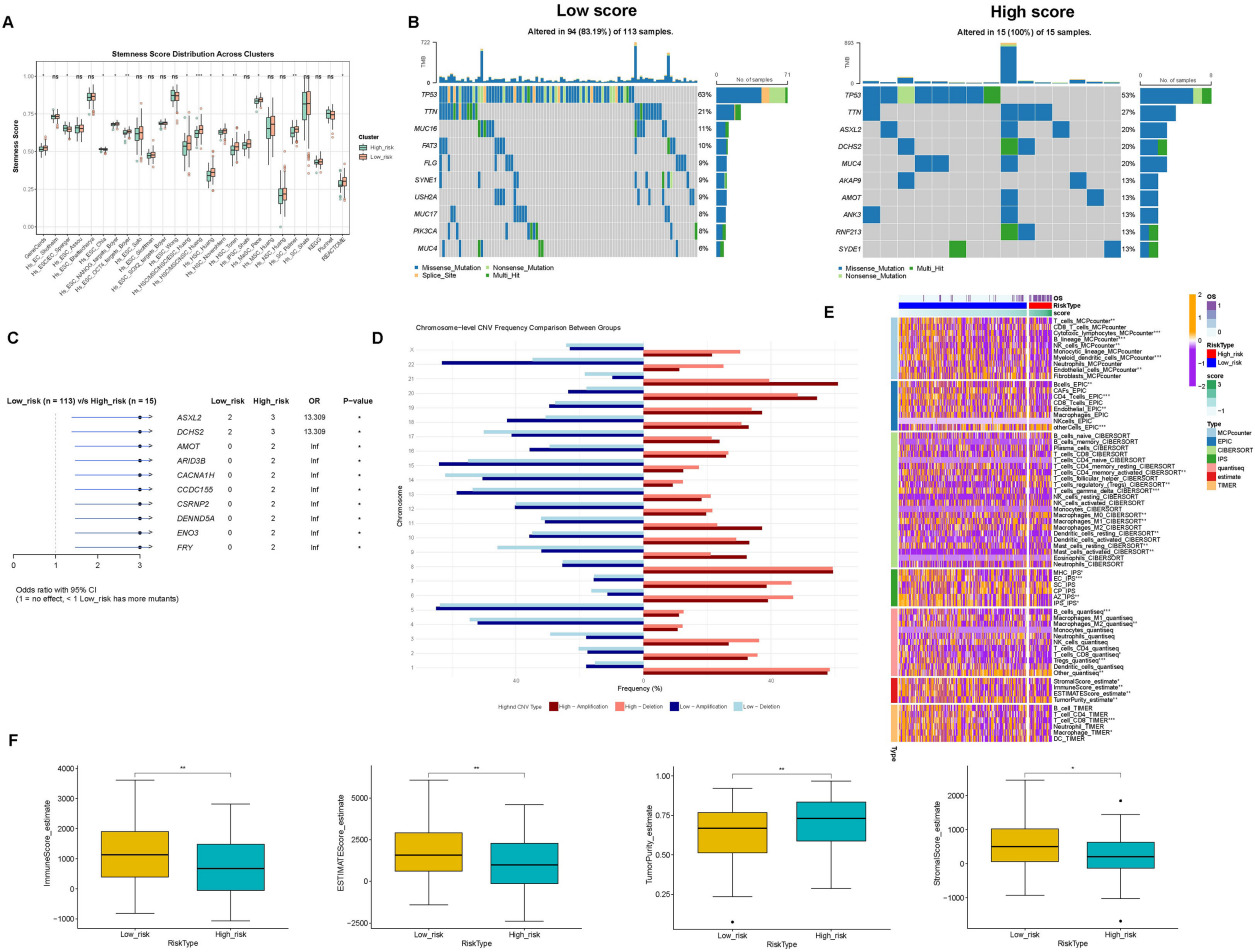
Integrated genomic and immune analyses identify biological divergence between risk groups. **(A)** Comparison of stemness scores between high- and low-risk groups. **(B)** Somatic mutation landscapes were generated for high- and low-risk groups. **(C)** Differential mutation analysis between high- and low-risk groups. **(D)** Chromosome-level CNV mutation frequency across groups, depicting copy-number gain/loss frequencies for each chromosomal segment. **(E)** Heatmap showing associations between the RSF risk score and immune cell infiltration. **(F)** Box plots comparing ESTIMATE metrics, including ESTIMATE Score, Immune Score, Stromal Score, and Tumor Purity, between the high- and low-risk groups. ns (p > 0.05), * (p < 0.05), ** (p < 0.01), *** (p < 0.001).

Further multi-omic profiling revealed that while the low-risk group exhibited higher stemness scores (**Figure 8A**), the high-risk group was characterized by pervasive genomic instability, including extensive somatic mutations (e.g., ASXL2, DCHS2) and severe chromosomal copy number variations (CNVs) (**Figures 8B–D**). Multi-method immune analysis integration confirmed a highly active immune state in the low-risk group (**Figure 8E**; **Supplementary Figure 9**), whereas ESTIMATE analysis showed the high-risk group possessed higher tumor purity but profoundly suppressed immune and stromal scores (**Figure 8F**).

**Figure 9:**
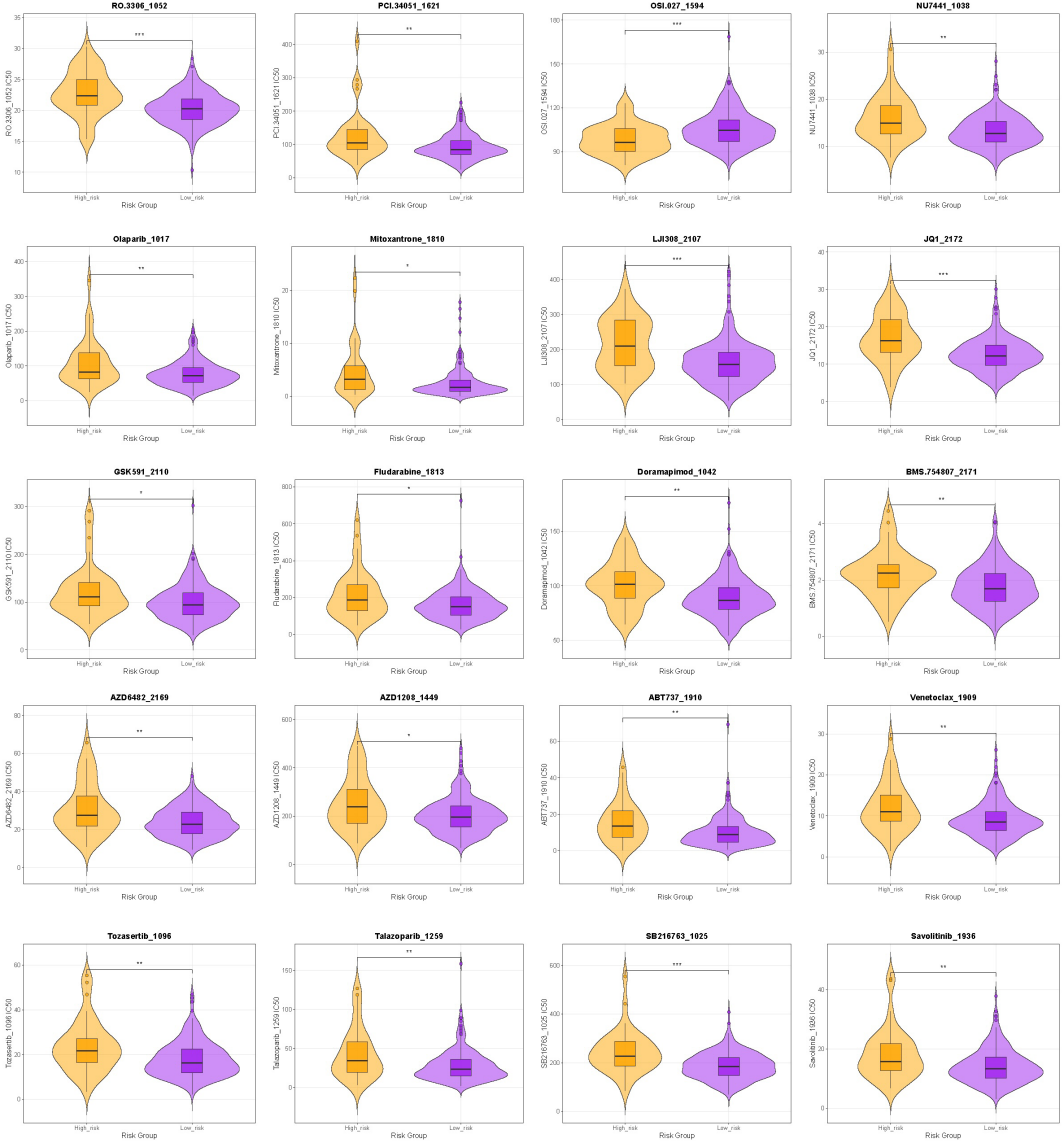
Drug sensitivity differences between risk score groups. IC50 values for multiple anticancer drugs were predicted per sample using the oncoPredict R package. Differences between high- and low-risk groups are compared, with the top 20 drugs ranked by the smallest P values displayed. ns (p > 0.05), * (p < 0.05), ** (p < 0.01), *** (p < 0.001).

To explore therapeutic implications, we estimated drug response using predicted IC50 values and compared them between risk groups. Overall, the high-risk group exhibited higher predicted IC50 values for most compounds, suggesting reduced sensitivity to multiple therapeutic classes (**Figure 9**). Notably, differences were observed across agents targeting cell-cycle control, epigenetic regulation, and DNA damage response pathways, indicating potential cross-resistance features in high-risk tumors.

## Discussion

Despite radiotherapy remaining a cornerstone of TNBC management, inter- and intratumoral heterogeneity profoundly limits consistent clinical benefits (14, 30, 31). Overcoming the limitations of single-omic approaches, our study utilized a multi-dimensional framework, integrating scRNA-seq, bulk cohorts, and machine learning—to decode the cellular and molecular determinants of TNBC radioresistance. We identified a 34-gene RSF prognostic model and, importantly, prioritized RAD51 through an unbiased, data-driven pipeline and validated it as a key driver of radioresistance and a promising therapeutic target, providing future treatment strategies for precision radiotherapy.

At the single-cell level, we identified proliferative tumor cells, monocyte/macrophage populations, and fibroblasts as major contributors to higher resistance scores. Their enrichment and spatial clustering support a niche-like architecture in which tumor cell repair/proliferation programs coexist with immunosuppressive and stromal remodeling signals (19, 32, 33). These observations reveal that radioresistance is shaped not only by intrinsic DNA damage responses but also by multicellular cooperation within the TME, which can restrict effective antitumor immunity and sustain survival signaling after irradiation (34–36).

Expanding to clinical cohorts, bulk RNA-seq defined two distinct molecular subtypes. Cluster A, characterized by metabolic reprogramming, high stemness, and genomic instability (high tumor mutational burden (TMB)), exhibited a highly resistant phenotype and poor survival. Conversely, Cluster B represented an “immune-hot” and radiosensitive profile. This dichotomy systematically links radioresistance to stemness and immune desertification, offering a biological rationale for subtype-specific combination therapies.

Building on these biological insights, we developed a 34-gene RSF model that showed robust performance across the discovery cohort and four independent external cohorts. Crucially, unlike single-gene evaluations that are susceptible to intratumoral heterogeneity, this multi-gene signature functions as a multidimensional gauge. In addition to risk stratification, the model captured key features of the TME: high-risk patients exhibited significantly diminished immune infiltration and higher tumor purity, and uniquely predicted multi-drug sensitivities (correlating with elevated predicted IC50 values for multiple chemotherapeutics). These patterns are consistent with enhanced intrinsic survival capacity and DNA damage–repair activity, which may contribute to broad treatment resistance (37–40). Clinically, this suggests that simply increasing radiation dose is unlikely to yield durable benefit in high-risk disease; rational combinations that inhibit DNA repair and/or metabolic vulnerabilities may be required to improve therapeutic response.

In order to discover more valuable therapeutic targets, we integrated model importance with DepMap dependency data, which nominated RAD51 as a leading candidate. Although RAD51 is a well-established mediator of homologous recombination with known prognostic relevance in breast cancer (41–45), our study extends beyond reaffirming its intrinsic DNA-repair capacity. Through an unbiased, top-down multi-omics pipeline, RAD51 independently emerged as a core dependency for TNBC radioresistance. This data-driven convergence not only validates the robustness of our computational framework but also critically maps RAD51 within a specific, immune-cold microenvironment. Consequently, our findings elevate RAD51 from a solitary DNA-repair mediator to a key target within multi-dimensional stratification strategy for high-risk patients. We then experimentally substantiated this dependency across multiple assays. *In vitro*, RAD51 knockdown markedly sensitized TNBC cells to irradiation by exacerbating unrepaired DNA damage (γ-H2AX accumulation), triggering sustained G2/M phase arrest, and driving apoptosis. *In vivo* models further demonstrated that RAD51 silencing synergized remarkably with radiotherapy to suppress tumor growth. Notably, beyond intrinsic tumor cell cytotoxicity, our findings indicate that the combination modulates the local immune microenvironment. This treatment enhanced CD8^+^ T cell infiltration and promoted a shift in macrophage polarization toward an anti-tumor M1 state. Additionally, the observed adaptive upregulation of PD-L1 expression provides a biological rationale for potentially integrating RAD51 inhibition and radiotherapy with immune checkpoint blockade in future translational studies.

While our integrative multi-omics framework provides comprehensive insights into TNBC radioresistance, several limitations should be acknowledged. Although the model was validated in four independent external cohorts, these were retrospective and partly comprised non-TNBC breast cancer samples; prospective validation in large-scale, dedicated TNBC cohorts is warranted to further substantiate clinical applicability. Furthermore, although H&E staining of major organs revealed no overt histopathological abnormalities, indicating general *in vivo* tolerability of the combination strategy, we did not perform detailed toxicological or selectivity profiling in normal cells and tissues. Systematic evaluation of the therapeutic window and potential normal-tissue toxicities of pharmacological RAD51 inhibitors combined with radiotherapy will therefore be an essential prerequisite for clinical translation.

Collectively, this study delineates the landscape of TNBC radioresistance across cellular and molecular dimensions, establishing a robust RSF-based risk stratification framework with potential clinical utility. Through an unbiased, data-driven strategy and rigorous experimental validation, we identify RAD51 as a fundamental driver of this resistant phenotype, and further show that its inhibition reprograms the immunosuppressive microenvironment. These findings highlight RAD51 inhibition as a promising radiosensitizing strategy to overcome therapeutic resistance, particularly for patients with high-risk, immune-cold tumors, and support its further evaluation in combination with immune checkpoint blockade.

## Conclusion

In conclusion, this study establishes a comprehensive multi-omics framework that decodes the cellular heterogeneity and molecular drivers of TNBC radioresistance. We developed a robust machine-learning prognostic model that enables risk stratification and stratifies tumors into immune-active and immune-cold phenotypes. Through an unbiased, data-driven strategy, we prioritized and experimentally validated RAD51 as a key driver of radioresistance. Its inhibition sensitizes TNBC to radiotherapy by exacerbating unrepaired DNA damage and remodeling the immunosuppressive microenvironment. Together, these findings bridge single-cell insights with clinical applicability, offering a translatable rationale for personalized combinatorial strategies—including the integration of immune checkpoint blockade— to overcome radioresistance in high-risk, immune-cold TNBC.

## Data Availability

The original contributions presented in the study are included in the article/**Supplementary Material**. Further inquiries can be directed to the corresponding author.
