# Supplementary material for: Risk stratification and multi-omics reveal RAD51 inhibition as a radiosensitizing strategy in triple-negative breast cancer
Source: Front Immunol. 2026 Jul 28;17:1866358. doi: 10.3389/fimmu.2026.1866358 (PMC13457375; doi:10.3389/fimmu.2026.1866358)
Supplement: Supplementary file 1 [file SupplementaryFile1.docx]

**Supplemental figure1**

**Fig. S1. Correlation of single- cell radiotherapy resistance scores with Hallmark Pathways.**

Hallmark pathways were downloaded from the MSigDB database, and single-cell enrichment scores were computed using the GSVA R package for both radiotherapy resistance–related gene sets and the Hallmark pathways. Correlations between radiotherapy resistance scores and Hallmark pathway scores across cells were then calculated, and the resulting correlation matrix was visualized as a heatmap.

**Supplemental figure 2**

**Fig. S2. Comparing differences between High- and Low-Scoring Groups based on a comprehensive analysis of three algorithms**

LogFC, odds ratio (OR), and direct difference (direct_diff) are all standardized using Z scores, and then summed to obtain a composite score (composite_score). These three methods are combined to reflect their comprehensive ranking and contribution.

**Supplemental figure 3**

**Fig. S3. Information-Flow analysis of High- vs. Low-Scoring Groups**

A bar chart visualization of Information-Flow Metrics for Different Genes, with red and cyan indicating the “High” and “Low” states, respectively.

**Supplemental figure 4**

**Fig. S4. Unsupervised Clustering of radiotherapy resistance-related genes**

Unsupervised consensus clustering of the 34 genes selected by Cox regression was performed with the R package ConsensusClusterPlus, identifying a two-subtype solution as optimal.

**Supplemental figure 5**

**Fig. S5. Enrichment analysis of Cluster A and Cluster B**

Pathways from the MSigDB Biocarta, Hallmark, KEGG, WikiPathways, and Reactome collections were downloaded and scored using the GSVA R package. Pathway differences between the two subtypes were assessed, revealing marked distinctions. And the result was visualized as a heatmap.

**Supplemental figure 6**

**Fig. S6. RFS model predicts prognosis and stratifies clinical risk.**

A. Kaplan–Meier survival curves in the TCGA and GSE58812 cohort stratified by RSF risk score. **B**. Kaplan–Meier survival curves in the four independent GEO cohorts stratified by RSF risk score. **C.** Univariable Cox regression of the RSF risk score and clinical covariates. **D.** Multivariable Cox regression adjusting for clinical covariates.

**Supplemental figure 7**

**Fig. S7. Enrichment analysis on score**

**A, B.** Heatmaps of the top 50 genes positively and negatively correlated with the model score across all samples. **C-E.** GSEA (clusterProfiler) of the genome ranked by score–gene correlation, showing the top 20 enriched GO, KEGG and Reactome terms.

**Supplemental figure 8**

**Fig. S8. Experimental validation of RAD51 as a core driver of radioresistance**

**A.** H-score of TNBC surgical specimens (n=20) post-adjuvant RT. Good responders: TRG 0–3; Poor responders: TRG 4–5. **B, C.** Representative images and quantification of EDU staining in 4T1 and MDA-MB-231 tumor cells. Scale bars: 20 μm. **D.** Representative H&E staining of major organs (heart, liver, spleen, lung, and kidney) from each treatment group. cale bars: 200 μm. **E.** Quantifications of CD8^+^, CD206^+^, and CD86^+^ cells infiltration in tumors. **F.** Western blot analysis of PD-L1 protein expression in tumor tissues from each treatment group, with GAPDH as the loading control. **Statistical analysis:** Data are presented as mean±SEM. Significance was assessed by Student’s t-test (A) and one-way ANOVA (B, C). *P < 0.05; **P < 0.01; ***P < 0.001; ****P < 0.0001.

**Supplemental figure 9**

**Fig. S9. Enrichment analysis of High- and Low-Scoring Groups**

Heatmap depicting the relationships between model scores and immune-related genes.

**Supplementary Table S1**

| **Gene** | **Importance** |
| --- | --- |
| **EIF4EBP1** | 0.055153493 |
| **SPP1** | 0.037403276 |
| **WNT2B** | 0.022980392 |
| **SERPINE1** | 0.020909215 |
| **PAG1** | 0.009610664 |
| **PGK1** | 0.009302817 |
| **HMOX1** | 0.009115164 |
| **LHX3** | 0.007874238 |
| **PKM** | 0.007175287 |
| **IAPP** | 0.007076486 |
| **ITGA6** | 0.006376344 |
| **RAD51** | 0.006143314 |
| **IDH2** | 0.005050599 |
| **CD274** | 0.002936199 |
| **FAS** | 0.001717945 |
| **BSG** | 0.000939503 |
| **ABCB1** | 0.000936044 |
| **CA9** | 0.000911055 |
| **ASS1** | 0.000354275 |
| **ATM** | -0.000833508 |
| **NFE2L2** | -0.001033181 |
| **VEGFA** | -0.001927728 |
| **NOX4** | -0.002729028 |
| **DRAM1** | -0.003055787 |
| **AKT1** | -0.003372 |
| **BANP** | -0.003691104 |
| **ALDOA** | -0.004263984 |
| **ERAP1** | -0.004464185 |
| **CARM1** | -0.006154305 |
| **EIF2AK2** | -0.006247132 |
| **PSMB8** | -0.006288875 |
| **TOB1** | -0.006748513 |
